# Transcutaneous auricular vagus nerve stimulation, tolerability, and the Alpha/Theta EEG ratio: a controlled pilot study relevant to emotion regulation

**DOI:** 10.3389/fpsyt.2026.1912977

**Published:** 2026-09-23

**Authors:** Seyedeh Zeinab Molaeizadeh, Aitor Aritzeta, Weronika Górska, Seyyedeh Fatemeh Molaeezadeh

**Affiliations:** 1 Department of Psychology, University of the Basque Country (UPV/EHU), San Sebastian, Spain; 2 Department of Chemistry, Warsaw University of Technology, Warsaw, Poland; 3 Department of Electrical and Computer Engineering, Jundi-Shapur University of Technology, Dezful, Iran

**Keywords:** Alpha/Theta ratio, EEG biomarkers, emotion regulation, stimulation intensity, tolerability, transcutaneous vagus nerve stimulation

## Abstract

**Background:**

Anxiety and depression are both characterised by difficulties in emotion regulation, associated with altered cortical oscillatory activity. Transcutaneous auricular vagus nerve stimulation (taVNS) may modulate these dynamics, but few studies have examined stimulation intensity alongside both tolerability and EEG outcomes.

**Methods:**

In this controlled pilot, ten healthy female adults (21.1 ± 1.8 years) were randomized to taVNS (n = 5) or a waitlist control (n = 5). The taVNS group completed eight 30-minute left tragus sessions over four weeks (20 Hz, 200 μs, 14–25 mA, individually titrated), with Alpha/Theta and Theta/Beta ratios derived from resting-state EEG at Cz. We applied False Discovery Rate (FDR) correction across the exploratory comparisons.

**Results:**

Protocol completion was 100% across 40 sessions. Of 39 stimulation-related observations, 29 were tolerability or adverse-event symptoms, predominantly CTCAE Grade 1 (86.2%); 10 were self-reported sensations of pulse change, recorded separately. None was serious. Within-group change in the taVNS group was not significant (exact p = .063 for both ratios). Stimulation intensity did not show a positive dose-response. Between-group comparisons of change scores favoured taVNS for both ratios (both U = 25, exact p = .008; Hodges-Lehmann median difference 0.56, 95% CI [0.12, 0.90] for Alpha/Theta) and remained significant after FDR correction.

**Conclusion:**

The protocol was completed by all five stimulated participants without serious adverse events under individual titration to tolerability (14–25 mA). We found preliminary effects on an EEG index relevant to emotion regulation, providing a quantitative basis for powering sham-controlled trials in clinical emotional dysregulation.

## 1 Introduction

EEG spectral dynamics offer a non-invasive window into cortical excitability shifts relevant to emotion regulation. Unlike single-band measures, band-power ratios capture how competing neural states balance. The Alpha/Theta ratio (8–13 Hz/4–8 Hz) is one well-studied example. It tracks relaxed alertness and inward attention, and its change is documented across neurofeedback studies in clinical and healthy groups (1–3). When alpha dominates theta, the profile corresponds to a calm, low-arousal cortical state. When theta dominates, arousal increases and attention is less well sustained, a pattern reported in anxiety and depression (4, 5). The Theta/Beta ratio (4–8 Hz/13–30 Hz) has been examined in a related context. It has been interpreted as an index of the balance between top-down (prefrontal/executive) control and bottom-up processing, with higher values reported in affective and attentional conditions (6). Evidence linking this ratio to arousal per se is inconsistent. Because higher values are usually reported as unfavourable, we did not have a directional hypothesis for this ratio and treat it as an exploratory secondary outcome; we do not interpret an increase as an improvement. We selected the Alpha/Theta ratio because it is the parameter directly targeted in Alpha/Theta neurofeedback protocols, which makes the present findings comparable with that literature; we do not treat it as a validated or specific index of emotion-regulatory capacity. Few controlled studies have tested whether neuromodulation can shift these ratios.

taVNS is delivered through skin electrodes at the tragus or cymba conchae, activating vagal afferents that carry the signal through the nucleus tractus solitarius to the locus coeruleus (7). Norepinephrine then spreads across prefrontal and limbic regions handling emotional appraisal and control (8). taVNS also modulates GABAergic interneurons, which set the inhibitory tone behind cortical excitability (9). taVNS has also been reported to reduce sympathetic nerve activity (10). Autonomic and cortical measures are not interchangeable, however: EEG-HRV coupling is heterogeneous across tasks and states, and no autonomic measures were recorded in this study. Sharon et al. (11) found taVNS reduced alpha oscillations alongside pupil dilation. Keute et al. (12) reported stronger frontal midline theta during inhibitory control. Konjusha et al. (13) saw lower prefrontal alpha activity during conflict monitoring. This has prompted interest in taVNS for emotional disorders, with early evidence in major depressive disorder (14). Reviews report consistent rises in theta and delta power, though alpha effects vary (6).

Two gaps limit translation to clinical settings. First, most controlled studies have looked at single frequency bands rather than composite ratios. Ratios map more directly onto neurofeedback protocols used for emotion regulation (5, 15). Second, few taVNS-EEG studies use healthy, stable adults (6). Such adults provide a baseline not confounded by psychopathology. This pilot addresses both gaps, testing whether a structured taVNS protocol shifts these ratios in healthy adults. Adverse events were tracked alongside intensity to inform safe parameters (16), and effect sizes estimated to power future trials in clinical emotional dysregulation (17). Only women were recruited, to limit sex-related variance in vagal tone and autonomic reactivity (18, 19). EEG was recorded at Cz, a standard midline central site close to frontal-midline theta generators (3).

## 2 Method

### 2.1 Study design

This controlled between-group pilot tested how a structured taVNS protocol affects EEG ratio markers in healthy adults. Ten participants were randomized 1:1 to taVNS (n = 5) or waitlist control (n = 5) using computer-generated sequences with concealed allocation in sealed envelopes. EEG was recorded at baseline and one week after the eighth session, at the same time of day, so that any change would reflect stable adaptation rather than acute post-session fluctuation. A research assistant blind to group allocation performed the ratio analysis. The University of the Basque Country Ethics Committee (M10-2020-318) approved all procedures, and each participant gave written informed consent under the Declaration of Helsinki.

Data from this participant cohort have been reported elsewhere with different research questions: a three-arm comparison of taVNS and neurofeedback (20) and a four-arm study including salivary cortisol and psychological outcomes (21). The present report is the only one to address stimulation tolerability, the Theta/Beta ratio, and the relationship between stimulation intensity and EEG change.

The study was not prospectively registered and no analysis plan was lodged in advance. The primary focus was feasibility and tolerability, with Alpha/Theta as the primary neurophysiological outcome and Theta/Beta as secondary. The between-group comparison, dose-response correlations, and FDR family were decided after data inspection and are exploratory. Participants were recruited through in-class announcements in undergraduate psychology courses, without compensation, within one academic year. Progression criteria were not defined in advance, and the study is reported as a small randomized feasibility study rather than to the full CONSORT pilot extension.

### 2.2 Participants

Ten healthy female students (mean age 21.1 ± 1.8 years) took part. Eligibility required age 18–24, no neurological, psychiatric, or cardiovascular disorder, no psychotropic medication, and normal sensory function. Exclusions were pregnancy, implanted cardiac device, seizure history, and ear abnormalities preventing placement.

Recruiting only women controlled for sex-related variance in vagal tone, autonomic reactivity, and resting EEG (18, 19), and gave a uniform baseline before moving to clinical groups. The sample was set at n = 5 per group on feasibility grounds, aiming for effect-size estimates rather than formal power (17).

### 2.3 Intervention protocol

The taVNS group received eight 30-minute sessions over four weeks, two per week with at least 48 hours between them, using the Parasym AVNT device (Parasym Scientific Ltd., London, UK). Electrodes were placed on the left tragus to target the auricular branch of the vagus nerve. Participants rested with eyes open and kept still. Settings were fixed at 200 μs and 20 Hz, with intensity titrated individually. Titration was repeated each session: the device started at its minimum (11 mA) and rose in 1–2 mA steps until the participant reported clear tingling at the site, then dropped to the highest comfortable perceptible level if unpleasant. Delivered intensities ranged from 14 to 25 mA. Per-participant mean intensity ranged from 16 to 22 mA. All sessions were supervised to keep placement consistent. Control participants completed only the baseline and post-intervention EEG recordings and received no intervention.

### 2.4 EEG recording and spectral analysis

EEG was recorded with a two-channel Vilistus system (Durham Systems Management, UK), with the active electrode at Cz, ground at the left mastoid (A1), and reference at the right mastoid (A2), per the international 10–20 system. Cz was pre-specified as the recording site, consistent with its standard use in Alpha/Theta protocols. It lies close to frontal-midline theta generators (3), whereas alpha is maximal over posterior regions. The central Alpha/Theta ratio is therefore best read as a practical composite index at a single midline site rather than a pure reflection of each band’s generators. Each resting recording lasted five minutes.

Spectral analysis used Fast Fourier Transform in the Vilistus software. Spectral power was computed for theta (4–8 Hz), alpha (8–13 Hz), and beta (13–30 Hz). The Alpha/Theta ratio was calculated as alpha absolute power divided by theta absolute power, and the Theta/Beta ratio as theta absolute power divided by beta absolute power. Mean ratios across artifact-free epochs were carried forward to the statistical analysis.

Signals were sampled at 512 Hz with band-pass filtering at 0.5–40 Hz. Scalp sites were abraded and electrodes affixed with conductive paste, impedances below 5 kΩ. Participants reclined in a quiet, dimly lit room, eyes closed, still and relaxed but awake. Pre-recording restrictions on food, caffeine, alcohol, and exercise applied to salivary sampling in the parent study but not to the EEG sessions; sleep, nicotine, medication, hormonal contraceptive use, and menstrual-cycle phase were not recorded. Artifact rejection was automatic; the thresholds, epoch length, retained-epoch count, proportion of data removed, FFT window and overlap, and spectral resolution are not exposed by the software. The assistant performing the ratio analysis was blind to allocation. These constraints limit exact replication.

### 2.5 Adverse event monitoring

After each taVNS session, participants completed an adverse event checklist adapted from Kim et al. (16), grading severity with the Common Terminology Criteria for Adverse Events (CTCAE v5.0) as Grade 1 (mild), Grade 2 (moderate), or Grade 3 (severe); absent symptoms were recorded as 0. The form also recorded relatedness to stimulation, resolution by session end, and action taken. The form covered itching or burning at the site, redness, headache, dizziness, and perceived heart-rate changes, drawn from prior taVNS safety reviews (16, 22). It was assembled for this study, has not been separately validated, and is reproduced in full in **Supplementary Appendix 1**. For each item participants recorded occurrence and severity, and indicated whether the symptom resolved by the end of the session or persisted beyond the visit. Study personnel reviewed each completed checklist and followed up verbally on any reported symptom. Predefined stopping rules specified discontinuation and referral for persistent moderate-to-severe symptoms requiring medical attention; none occurred. Events were classified using **Supplementary Table 1**, which defines expected stimulation sensations, tolerability symptoms, clinically relevant adverse events, and autonomic safety observations. We recorded unwanted cutaneous sensations such as itching and burning at each session, separately from the expected tingling used to titrate intensity. For cardiovascular responses, participants reported any perceived pulse changes, which we checked by radial pulse palpation. This provided a basic safety screen only, so perceived pulse changes are reported descriptively as autonomic safety observations rather than as adverse events.

### 2.6 Statistical analysis

Analyses used IBM SPSS Statistics 27. Given the small sample and non-normality (Shapiro-Wilk), pre-post values were compared within groups using Wilcoxon signed-rank tests. All tests used exact p-values, since asymptotic approximations are unreliable at n = 5; we report the signed-rank statistic W and the number of non-zero pairs. Paired effect size is the matched-pairs rank-biserial correlation (difference between positive and negative signed-rank sums divided by their total). The two-tailed threshold was p < 0.05. Groups were compared directly on change scores (Δ = post − pre) using Mann-Whitney U tests with exact p-values, reporting the Hodges-Lehmann median difference with an exact 95% confidence interval. These comparisons are exploratory given the sample size. To limit Type I error, we applied Benjamini-Hochberg False Discovery Rate correction (23) across six comparisons (four within-group and two between-group). The two between-group comparisons remained significant after correction; no within-group comparison did. We therefore treat the within-group p-values as exploratory and report effect sizes as the main output for designing properly powered trials (24, 25).

## 3 Results

Ten female participants (mean age 21.1 ± 1.8 years) were assigned at random to taVNS (n = 5) or waitlist control (n = 5). Given the small sample and the pilot design, the findings are exploratory and hypothesis-generating.

### 3.1 Feasibility and protocol completion

All ten participants completed the protocol with 100% retention, all 40 scheduled sessions were delivered without premature discontinuation, and no participant withdrew for tolerability reasons. The protocol was therefore acceptable and feasible to deliver.

### 3.2 Stimulation-related observations and tolerability profile

#### 3.2.1 Overall stimulation-related observations

Across 40 sessions, 39 stimulation-related observations were recorded: 29 tolerability or adverse-event observations (27 cutaneous, one headache, one dizziness) and 10 self-reported sensations of pulse change, recorded separately since no quantitative heart-rate values were measured. At least one observation occurred in 24 of 40 sessions (60.0%) and in all five participants; pulse-change reports in 10 sessions (25.0%) in three participants. No event required medical attention and none led to discontinuation or withdrawal. Events were classed using **Supplementary Table 1**. Among the 29 tolerability and adverse-event observations, severity was confined to CTCAE Grade 1 (25, 86.2%) and Grade 2 (four, 13.8%, all skin sensations in VE008). No Grade 3 event occurred, and all 10 pulse-change reports were Grade 1. No serious adverse events occurred. Observations ranged from 2 (RO807, SA974) to 18 (VE008); **Table 1** gives the breakdown.

**Table 1:** Participant-level stimulation-related observations (N = 5, taVNS group).

ID	Sessions with obs.	Mean intensity (mA)	Intensity range (mA)	Skin	Headache	Dizziness	Tolerability/AE subtotal	Pulse-change reports	Total obs.
AR833	7	18.6	14–22	7	0	0	7	2	9
VI207	7	21.9	15–25	7	0	1	8	0	8
RO807	2	16.1	14–18	1	0	0	1	1	2
VE008	7	19.9	15–24	10	1	0	11	7	18
SA974	1	19.5	15–23	2	0	0	2	0	2

Counts are session-level ratings above zero. The tolerability/AE subtotal covers skin, headache, and dizziness observations, all CTCAE Grade 1 or 2 (the four Grade 2 were skin sensations in VE008). Pulse-change reports are self-reported sensations of altered heart rate, recorded separately as no quantitative values were measured.

#### 3.2.2 Tolerability and safety observation categories

Cutaneous effects: Unwanted skin sensations, mainly itching and mild burning, were most common and reported by all five participants; they were brief and needed no treatment. The expected tingling used to set intensity was not counted.

Autonomic safety observations: Three participants (AR833, RO807, VE008) reported sensations of pulse change, 10 in total, checked by radial pulse palpation. None was persistent, associated with clinically relevant symptoms, or required intervention.

Other effects: One headache and one episode of dizziness, both mild.

#### 3.2.3 Intensity and event frequency

Frequency did not track intensity: the highest-intensity participant (VI207, 21.9 mA) reported 8, while the most affected (VE008, 18) was stimulated at a mean of 20 mA. Four of the five participants had mean intensities at or below 20 mA. The distribution of observations across participants is shown in **Supplementary Figure 1**. VE008 (mean 20 mA, maximum 24 mA) accounted for all four Grade 2 events; participant-level detail is in **Supplementary Table 2**.

The profile is consistent with reviews describing taVNS as low-risk (16, 26). This variation underlines the need for individual titration to tolerability.

### 3.3 Neurophysiological outcomes

**Table 2** summarizes the pre- and post-intervention EEG ratios. All p-values are exact. Benjamini-Hochberg correction (23) was applied across the six comparisons: the two between-group comparisons survived (adjusted p = .024), no within-group comparison did, so those are exploratory trends needing replication.

**Table 2:** Pre- and post-intervention Alpha/Theta and Theta/Beta ratios by group.

Group	Measure	Pre (M ± SD)	Post (M ± SD)	p-value	Statistic, effect size
taVNS	Alpha/Theta	1.63 ± 0.19	2.05 ± 0.32	0.063	W = 0, r = 1.00
	Theta/Beta	2.04 ± 0.36	2.63 ± 0.56	0.063	W = 0, r = 1.00
Control	Alpha/Theta	1.84 ± 0.30	1.71 ± 0.25	0.250	W = 1, r = −0.80
	Theta/Beta	2.55 ± 0.46	2.33 ± 0.56	0.625	W = 3, r = −0.40
Between-group	Alpha/Theta Δ	taVNS +0.42 ± 0.26	Control −0.13 ± 0.17	0.008	U = 25, r = 1.00
	Theta/Beta Δ	taVNS +0.59 ± 0.39	Control −0.22 ± 0.36	0.008	U = 25, r = 1.00

p-values are exact and uncorrected; after Benjamini-Hochberg correction across six comparisons only the two between-group comparisons remained significant (adjusted p = .024). Within-group rows give the Wilcoxon signed-rank statistic W and matched-pairs rank-biserial r; between-group rows compare change scores (Δ = post − pre) with Mann-Whitney U tests.

#### 3.3.1 Alpha/Theta ratio

In the taVNS group, the Alpha/Theta ratio increased from pre-intervention (M = 1.63, SD = 0.19) to post-intervention (M = 2.05, SD = 0.32), representing a 25.8% increase (W = 0, five non-zero pairs, exact p = 0.063, matched-pairs rank-biserial r = 1.00) (**Figure 1**). This within-group comparison was not significant and did not survive FDR correction; it should be read as exploratory. All five participants showed upward trajectories, with increases from roughly 8% to 42% (**Figure 2**).

**Figure 1:**
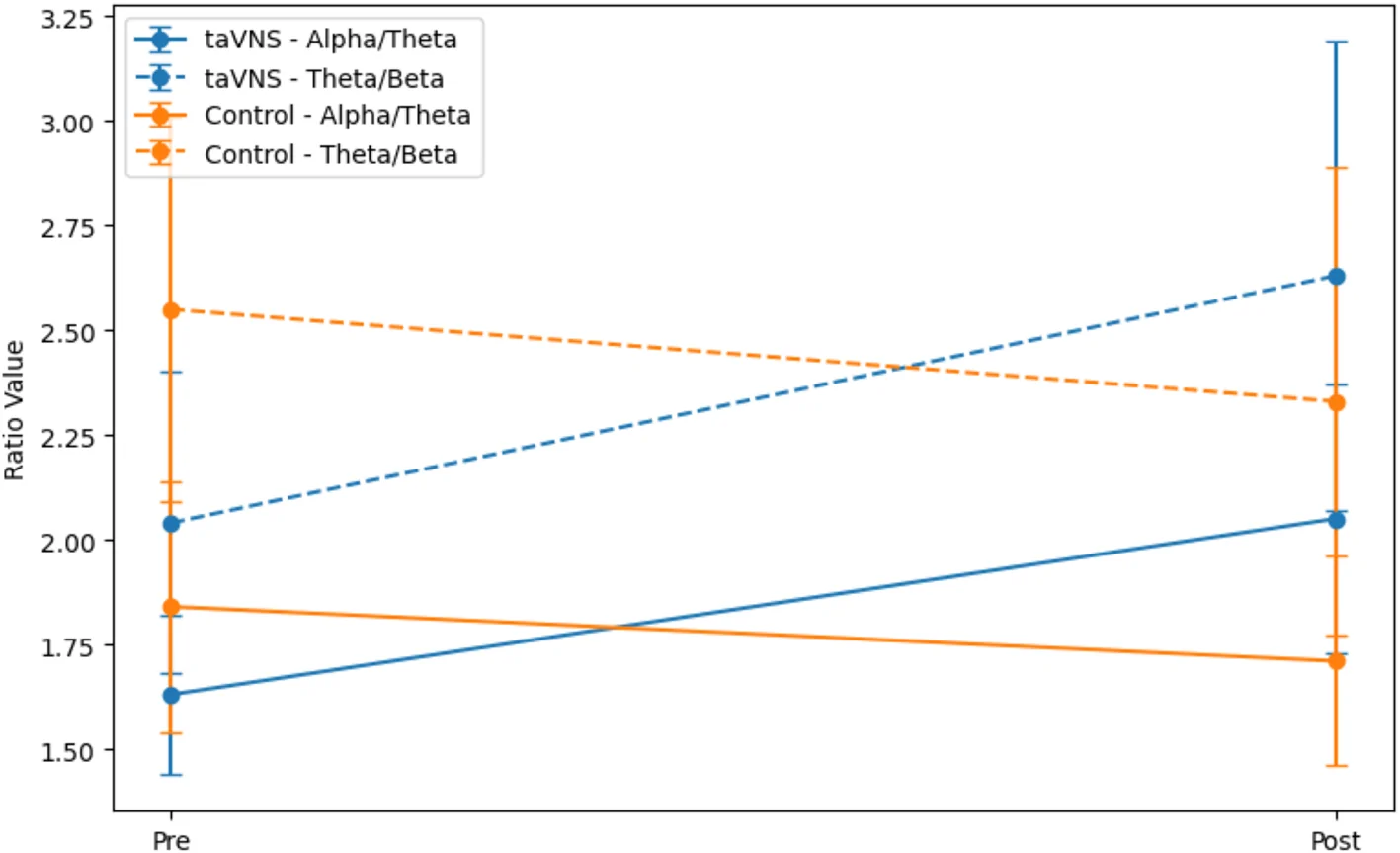
Pre-post changes in Alpha/Theta and Theta/Beta ratios. Group means with error bars showing ± 1 SD.

**Figure 2:**
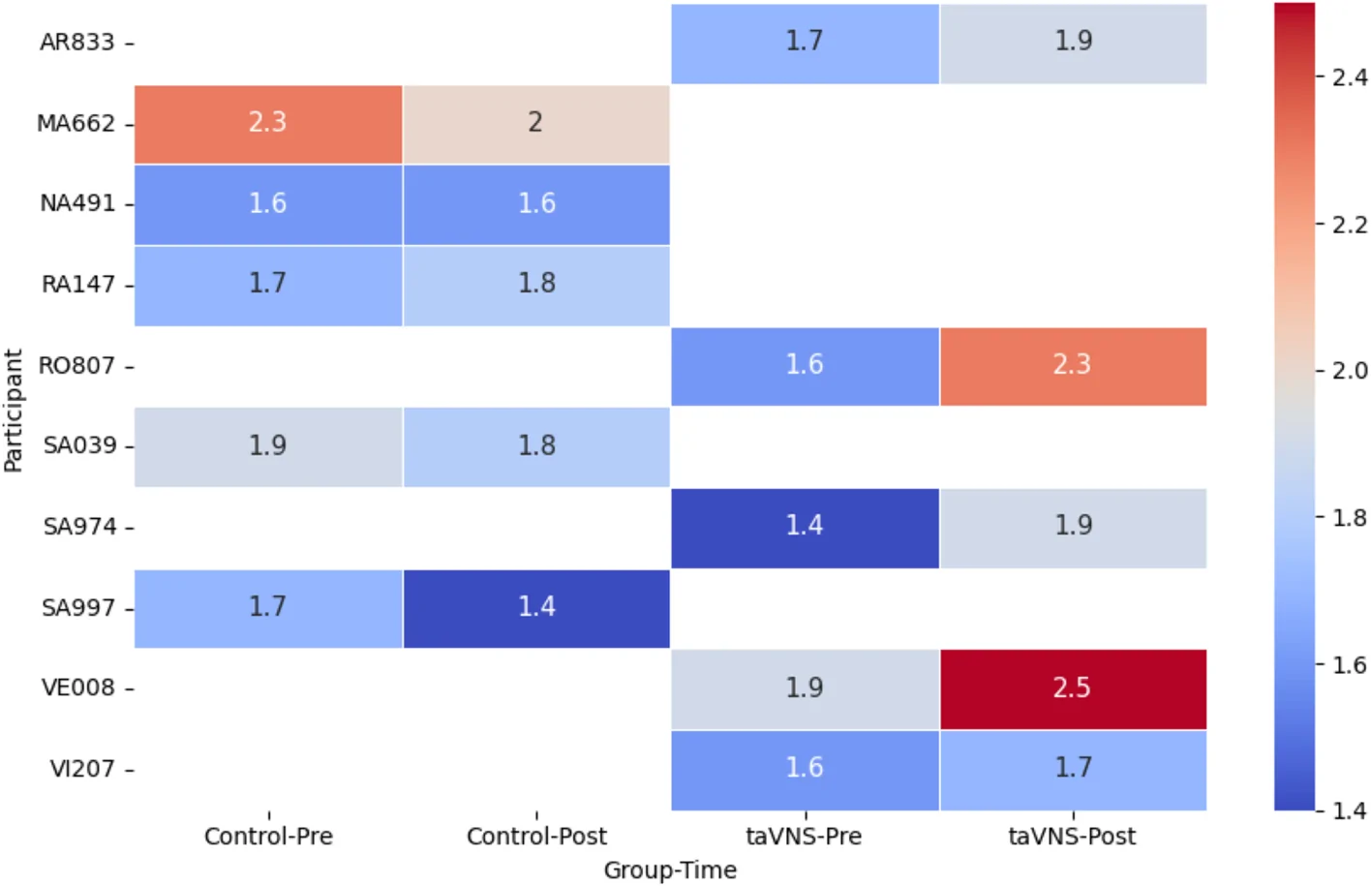
Individual trajectories for Alpha/Theta ratios. All five taVNS participants increased. Among controls, one increased slightly, one was unchanged, and three decreased; the control group mean decreased.

The control group changed little, decreasing slightly from pre (M = 1.84, SD = 0.30) to post (M = 1.71, SD = 0.25; W = 1, four non-zero pairs, exact p = 0.250, rank-biserial r = −0.80). With the between-group comparison in Section 3.3.3, this indicates the taVNS change is unlikely to reflect measurement noise alone.

#### 3.3.2 Theta/Beta ratio

The Theta/Beta ratio increased in the taVNS group from pre (M = 2.04, SD = 0.36) to post (M = 2.63, SD = 0.56), representing a 28.9% increase that did not reach significance (W = 0, five non-zero pairs, exact p = 0.063, rank-biserial r = 1.00). After FDR correction, this is exploratory only. The control group showed no meaningful change (pre: M = 2.55, SD = 0.46; post: M = 2.33, SD = 0.56; W = 3, four non-zero pairs, exact p = 0.625, rank-biserial r = −0.40) (**Supplementary Figure 2**).

The two ratios share theta power and are not independent, so we do not compare their effect sizes. Any claim of frequency specificity would require a direct statistical comparison with a prespecified hypothesis.

#### 3.3.3 Between-group comparison of change scores

Change scores (Δ = post − pre) differed between groups for both ratios. Alpha/Theta increased in the taVNS group (M = +0.42, SD = 0.26) and decreased slightly in controls (M = −0.13, SD = 0.17); U = 25, exact p = .008, r = 1.00. Theta/Beta showed the same pattern (taVNS M = +0.59, SD = 0.39; control M = −0.22, SD = 0.36; U = 25, exact p = .008, r = 1.00; Hodges-Lehmann median difference 0.85, 95% CI [0.14, 1.46]). All taVNS change scores exceeded all control change scores, and both comparisons survived Benjamini-Hochberg correction. The study is underpowered at five per group, and complete separation can overstate precision.

#### 3.3.4 Exploratory dose-response analysis

Spearman correlations between mean intensity and EEG change were not significant and gave no sign of a positive, intensity-dependent effect; if anything they were inverse (Alpha/Theta rs = −0.60; Theta/Beta rs = −0.30). With five participants these correlations are unstable, so no dose-response can be established in either direction.

## 4 Discussion

### 4.1 Oscillatory correlates of emotional regulation: interpreting the taVNS effect

The main finding of this pilot was that change scores in the Alpha/Theta ratio separated the two groups (U = 25, exact p = .008; Hodges-Lehmann median difference 0.56, 95% CI [0.12, 0.90]), alongside a 25.8% within-group rise in the taVNS group that was not itself significant (exact p = .063). The ratio reflects the balance between alpha, tied to relaxed inward processing, and theta, tied to arousal (1, 27). The ratio rose in all five taVNS participants while controls did not. This is a change in a resting EEG spectral ratio at a single midline site. With no behavioural, self-report, or autonomic outcomes included in the present analysis, the study cannot establish improved emotion regulation, reduced reactivity, or stronger top-down control. We do not interpret it as evidence of a change in autonomic or parasympathetic state, since no autonomic outcomes were included in the present analysis. This within-group comparison did not survive Benjamini-Hochberg correction across the six comparisons, although the corresponding between-group comparison did. Earlier single-band taVNS-EEG studies report effects on frontal midline theta (12), prefrontal alpha (13), and theta in a clinical sample (28), but give p-values rather than comparable effect sizes, so we cannot judge whether the present effect is larger. Whether composite ratios track taVNS effects more sensitively remains open. Answering it would require a head-to-head analysis in a powered trial (24). The Theta/Beta ratio shifted in the same direction, although this change was not statistically significant (28.9% increase, exact p = .063), which we report as an exploratory secondary outcome. Because higher Theta/Beta values are generally reported as unfavourable, we do not interpret this increase as improved regulation; its direction is inconsistent with the Introduction and remains unexplained. This contrasts with the lower cortical excitability that reviews link to vagal neuromodulation (6).

The likely pathway runs through ascending vagal afferents travelling via the nucleus tractus solitarius to the locus coeruleus, where norepinephrine shapes prefrontal and limbic excitability (7). taVNS also engages GABAergic interneurons, raising synchronized alpha and dampening beta-driven excitability (9, 11). This pathway is a hypothesis from prior literature, not a mechanism established here: we measured neither vagal engagement, norepinephrine, and GABA, nor autonomic activity or cortical connectivity. No dose-dependent relationship between intensity and Alpha/Theta change was found; the association was non-significant and, if anything, inverse. With five participants this is uninformative, and future work should map the intensity-response relationship using preset levels.

### 4.2 Safety profile and stimulation parameters

Across 40 sessions, 25 of 29 tolerability and adverse-event observations (86.2%) were CTCAE Grade 1 and four Grade 2, with no Grade 3 event and none serious, consistent with large systematic reviews (16, 26). Perceived pulse changes are treated as autonomic safety observations rather than adverse events, since modest subjective variation may be expected and no objective measurement was available. Because stimulation was titrated to a perceptual threshold, mild local sensations should be distinguished from clinically relevant events. Burden was not tied to intensity: the most affected participant averaged 20 mA while the highest-intensity participant reported few, indicating individual sensitivity rather than a threshold (22). The pulse-change reports were subjective observations checked by radial palpation; no heart-rate values were measured. This is less informative than the continuous ECG monitoring now standard for taVNS trials (28), so future studies should include objective cardiovascular recording, particularly as stimulation parameters can influence heart rate (29).

### 4.3 Limitations, clinical implications, and future directions

Several limitations constrain these findings. With five per group the study provides limited precision and was not powered for confirmatory inference: detecting a large effect (d = 0.8) at 80% power would need about 26 participants per group, or 17 for d = 1.0 (30). Pilot estimates of this size may also be unstable or inflated, so future trials should use a prespecified sample-size calculation based on a clinically meaningful effect rather than the present point estimates (24). We also had no active sham, which would stimulate a non-innervated ear site to preserve sensation and allow blinding (31, 32). Without it, expectancy effects cannot be separated from physiological ones (33), and a sham-controlled design would be needed to separate them. A single midline electrode at Cz cannot capture the network-level changes fMRI reveals after taVNS (34), nor the source-level connectivity high-density EEG would provide (35, 36). The eight-session protocol is also short; durable oscillatory change typically requires 15–20 sessions (5, 15). Finally, we recruited only women to reduce variance in vagal tone and autonomic reactivity (18, 19). We did not record menstrual-cycle phase or hormonal contraceptive use, both of which affect resting EEG and autonomic measures, so the extent to which this restriction reduced physiological variance is unknown. Findings should be generalized no further than healthy female university students aged approximately 18–24 years.

A change in a ratio also cannot show whether the numerator rose or the denominator fell, and theta appears in the denominator of Alpha/Theta and the numerator of Theta/Beta, so the two outcomes are mathematically coupled. The software computes each ratio as a dedicated channel, while the band amplitudes it exports derive from a separate dominant-frequency routine, so the ratios cannot be validly decomposed from the available data. Component-level analysis requires raw spectral output and is left to future work.

Despite these limitations, the data inform the design of a properly powered trial through procedures, retention, and immediate tolerability, though they cannot fix its effect-size or safety parameters. Depression and anxiety involve HPA-axis dysregulation, reduced heart rate variability, and disrupted prefrontal-amygdala connectivity (4, 37). This profile may respond to taVNS, as early work in major depressive disorder suggests (14, 38–40). Vagus nerve stimulation has also been explored for anxiety, though that evidence includes other modalities such as ultrasound (41), and taVNS has been applied with the same device family in field settings (42). Future work should use longer protocols (8–12 weeks), pair high-density EEG with fMRI, building on the BOLD findings reported for taVNS (34), and test whether taVNS added to psychological treatment yields extra benefit (43). Pilot estimates of this size are imprecise and likely inflated, so sample-size planning should draw on external evidence and the lower bound of our confidence intervals rather than these point estimates.

### 4.4 Conclusion

In this pilot, eight taVNS sessions were followed by an increase in the Alpha/Theta ratio in every stimulated participant, but not in controls. Change scores separated the two groups. With no behavioural or autonomic outcome measured, this is best described as a resting EEG spectral-ratio change potentially relevant to future emotion-regulation research. The within-group comparison was not significant and did not survive correction for multiple comparisons; the between-group comparison of change scores did. This suggests composite ratios may track vagal effects on cortical state more sensitively. The Theta/Beta ratio moved in parallel, though the within-group change was not significant (exact p = .063); the between-group comparison of change scores was significant (U = 25, exact p = .008). No dose-dependent link between intensity and EEG change was found, so optimal intensity remains an open question. Across 40 sessions, 39 events were recorded, all CTCAE Grade 1 or 2, none serious. Five participants cannot establish a safety profile; the defensible statement is that five participants completed the protocol without serious events. These results inform future powered, sham-controlled trials, particularly those extending the protocol to clinical groups with disrupted Alpha/Theta dynamics.

## Data Availability

De-identified individual data supporting the findings of this study, comprising EEG ratio values, stimulation intensities, and adverse-event records, are available from the corresponding author without undue reservation. Analysis syntax is available on request.
