# Supplementary material for: Transcutaneous auricular vagus nerve stimulation, tolerability, and the Alpha/Theta EEG ratio: a controlled pilot study relevant to emotion regulation
Source: Front Psychiatry. 2026 Sep 23;17:1912977. doi: 10.3389/fpsyt.2026.1912977 (PMC13643422; doi:10.3389/fpsyt.2026.1912977)
Supplement: Supplementary file 1 [file DataSheet1.docx]

**Supplementary Material**

*Transcutaneous Auricular Vagus Nerve Stimulation, Tolerability, and the Alpha/Theta EEG Ratio: A Controlled Pilot Study Relevant to Emotion Regulation*

**Supplementary Table 1.** Classification of stimulation-related sensations, tolerability symptoms, adverse events, and autonomic safety observations

| **Category** | **Examples** | **Timing** | **Reporting approach** |
| --- | --- | --- | --- |
| Expected stimulation-related sensations | Tingling, mild itching, pulsing, mild local sensation, perception of current | During stimulation only; resolves immediately or shortly after | Reported separately from adverse events unless distressing and persistent |
| Tolerability symptoms | Burning, uncomfortable ear sensation, skin irritation, unpleasant local discomfort, need to reduce intensity | During or shortly after stimulation | Reported as tolerability outcomes, especially if they require intensity adjustment |
| Clinically relevant adverse events | Headache, dizziness, nausea, persistent pain, persistent skin irritation, symptomatic or clinically concerning heart-rate changes, presyncope | During or after the session; may persist or require monitoring | Reported as adverse events with severity, duration, and action taken |
| Autonomic safety observations | Self-reported sensations of increased or decreased pulse without symptoms, verified by radial palpation only | During or shortly after stimulation | Reported separately from adverse events unless symptomatic, persistent, objectively abnormal, or requiring intervention |

*Note: A modest subjective variation in pulse may be expected during stimulation. Perceived heart-rate changes were therefore recorded as autonomic safety observations rather than cardiovascular adverse events, since no quantitative heart-rate values were obtained.*

**Supplementary Table 2.** Participant-level stimulation intensity, tolerability, and autonomic safety observations

| **ID** | **Mean intensity (mA)** | **Intensity range (mA)** | **Max tolerated (mA)** | **Sessions >20 mA** | **Expected sensations** | **Tolerability symptoms** | **Clinical AEs** | **Perceived HR changes** | **Total observations** |
| --- | --- | --- | --- | --- | --- | --- | --- | --- | --- |
| **AR833** | 18.6 | 14–22 | 22 | 1 | — | 7 | 0 | 2 | 9 |
| **VI207** | 21.9 | 15–25 | 25 | 4 | — | 7 | 1 | 0 | 8 |
| **RO807** | 16.1 | 14–18 | 18 | 0 | — | 1 | 0 | 1 | 2 |
| **VE008** | 19.9 | 15–24 | 24 | 1 | — | 10 | 1 | 7 | 18 |
| **SA974** | 19.5 | 15–23 | 23 | 1 | — | 2 | 0 | 0 | 2 |

*Note: Mean intensity = average session-level intensity across eight sessions. Intensity range = lowest to highest session-level intensity. Max tolerated = highest session-level intensity reached without discontinuation. Expected sensations (tingling) served as the titration target and were not logged as discrete observations (shown as —). Tolerability symptoms = skin events (itching or burning, redness). Clinical AEs = headache and dizziness. Perceived heart-rate changes were recorded separately as autonomic safety observations and were not classified as adverse events, since none was symptomatic, persistent, objectively abnormal, or required intervention. Counts are the number of session-level symptom ratings greater than zero.*

**Supplementary Figure 1.** Heatmap of cumulative stimulation-related observations by participant

*Note: Cell values are severity-weighted ratings summed across the eight sessions (the sum of per-session severity scores), so they may exceed the counts in Supplementary Table 2, which counts each session-level rating greater than zero as one observation. Cutaneous sensations dominate, while perceived heart-rate changes appear selectively in specific individuals.*

**Supplementary Figure 2.** Individual Theta/Beta ratios by group and time point

*Note: Inter-individual variability was greater than for Alpha/Theta. All five taVNS participants increased, whereas control participants showed minimal change.*

**Supplementary Appendix 1.** Adverse Event Questionnaire for taVNS

*Session-level form completed by each participant immediately after every stimulation session. The form was administered in Spanish; the version shown here is an English translation.*

**1. General Information**

| **Date** |  |
| --- | --- |
| **Participant code** |  |
| **Session** | ______ of 8 |
| **Stimulation parameters** | Intensity: ______ mA Frequency: ______ Hz |

**2. Adverse Events (graded according to CTCAE v5.0)**

| **Symptom** | **Grade 1 (Mild)** | **Grade 2 (Moderate)** | **Grade 3 (Severe)** | **Related to taVNS?** |
| --- | --- | --- | --- | --- |
| ***Skin*** |  |  |  |  |
| Itching / burning at the site | ☐ | ☐ | ☐ | Yes ☐ No ☐ |
| Redness | ☐ | ☐ | ☐ | Yes ☐ No ☐ |
| ***Neurological*** |  |  |  |  |
| Headache | ☐ | ☐ | ☐ | Yes ☐ No ☐ |
| Dizziness | ☐ | ☐ | ☐ | Yes ☐ No ☐ |
| ***Cardiorespiratory*** |  |  |  |  |
| Increased heart rate | ☐ | ☐ | ☐ | Yes ☐ No ☐ |
| Decreased heart rate | ☐ | ☐ | ☐ | Yes ☐ No ☐ |

**3. Symptom Course**

☐ Resolved by the end of the session	☐ Persisted beyond the laboratory visit

**4. Action Taken**

☐ Intensity reduced

☐ Stimulation temporarily interrupted

☐ Continued without change

☐ Other: ______________________________________

*Note: Severity was graded with the Common Terminology Criteria for Adverse Events (CTCAE) v5.0: Grade 1 mild, Grade 2 moderate, Grade 3 severe. Absent symptoms were recorded as 0. Expected stimulation sensations used to titrate intensity (tingling or prickling at the site) were the titration target and were not logged as discrete observations. Predefined stopping rules specified that persistent moderate-to-severe symptoms requiring medical attention would trigger discontinuation and clinical referral; no such event occurred. Heart-rate items record self-reported sensations of pulse change, verified by radial pulse palpation; no electrocardiographic or quantitative heart-rate values were obtained.*
